# The Coral Triangle Atlas: An Integrated Online Spatial Database System for Improving Coral Reef Management

**DOI:** 10.1371/journal.pone.0096332

**Published:** 2014-06-18

**Authors:** Annick Cros, Nurulhuda Ahamad Fatan, Alan White, Shwu Jiau Teoh, Stanley Tan, Christian Handayani, Charles Huang, Nate Peterson, Ruben Venegas Li, Hendra Yusran Siry, Ria Fitriana, Jamison Gove, Tomoko Acoba, Maurice Knight, Renerio Acosta, Neil Andrew, Doug Beare

**Affiliations:** 1 Indo-Pacific Division, The Nature Conservancy, Honolulu, Hawaii, United States; 2 Natural Resources Management, Worldfish, Bayan Lepas, Penang, Malaysia; 3 Coral Triangle Program, WWF-Indonesia, South Jakarta, Indonesia; 4 Coral Triangle Program, WWF-United States, Washington, DC, United States; 5 Indo-Pacific Division, The Nature Conservancy, Queensland, Australia; 6 Coordination and External Affairs, Coral Triangle Initiative on Coral Reefs and Food Security, Jakarta, Indonesia; 7 The Coral Triangle Center, Bali, Indonesia; 8 Joint Institute for Marine and Atmospheric Research, National Oceanic and Atmospheric Administration, Honolulu, Hawaii, United States; 9 Coral Triangle Support Partnership, WWF-Indonesia, Jakarta, Indonesia; 10 Regional Environment Office, United States Agency for International Development Regional Development Mission for Asia, Bangkok, Thailand; Universidade Federal do Rio de Janeiro, Brazil

## Abstract

In this paper we describe the construction of an online GIS database system, hosted by WorldFish, which stores bio-physical, ecological and socio-economic data for the ‘Coral Triangle Area’ in South-east Asia and the Pacific. The database has been built in partnership with all six (Timor-Leste, Malaysia, Indonesia, The Philippines, Solomon Islands and Papua New Guinea) of the Coral Triangle countries, and represents a valuable source of information for natural resource managers at the regional scale. Its utility is demonstrated using biophysical data, data summarising marine habitats, and data describing the extent of marine protected areas in the region.

## Introduction

**Figure 1:**
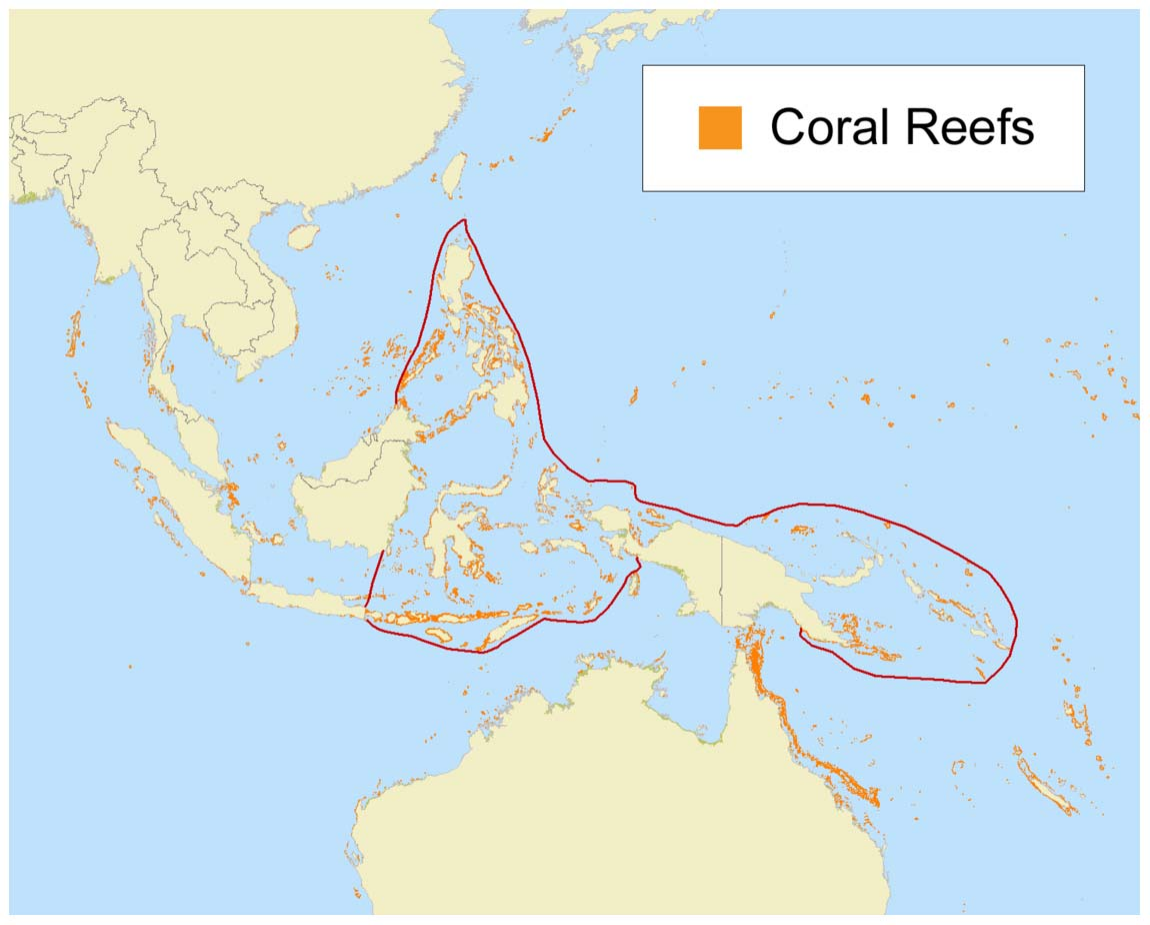
Map of the Coral Triangle region showing (orange polygons) the distribution of coral reefs and the boundaries of the Coral Triangle (red line) according to [12].

The Coral Triangle (CT) is located in South-east Asia and the Pacific, and encompasses the beautiful, tropical marine waters of Indonesia, Malaysia, Papua New Guinea, Philippines, Solomon Islands and Timor-Leste (The CT6, Fig. 1). It is recognized by the scientific community as *the* global centre of marine biological species diversity supporting [1], [2], for example, more than 605 species of reef-building corals; 15 of which are regional endemics [3]. This amounts to 76% of the global total species complement of corals, giving it the world's highest conservation priority [4]. It is estimated that more than 395 million people live in the Coral Triangle, 130 million of which directly depend on these resources for their livelihoods and well-being. Dependency on coastal and marine resources is especially true for those living in coastal communities [5] where, in some cases, up to 90% of protein uptake can be from fish [6]. The area attracts millions of tourists each year who take advantage of its pristine marine parks, nature reserves, famous dive sites, and beaches. The area is also an important nursery ground for both fish, and shellfish, which in turn support valuable commercial fisheries. The Coral Triangle is currently facing diverse threats connected with rapidly growing human populations, and associated economic development. Overfishing threatens the food security of its coastal communities [7], mangroves are being cleared for firewood, aquaculture and hotels, while the corals themselves are under threat from increasing sea temperatures [8], [9], sea level rise and ocean acidification [10]. Lack of effective marine spatial plans, and uncontrolled coastal development are the main threats to the Coral Triangle's reefs.

The area is particularly difficult to manage, however, because it falls under the remit of six national jurisdictions and many more sub-national jurisdictions and governance processes. In recognising this fundamental problem, the leaders of all six countries in the Coral Triangle (CT6) region came together to form the Coral Triangle Initiative for Coral Reefs, Fisheries and Food Security (CTI-CFF). Its express purpose is to safeguard the livelihoods of the communities that depend on its coastal resources: a key component of which is how to manage the exploitation of these resources sustainably.

The CTI-CFF, which was originally promoted by the President of Indonesia, Susilo Bambang Yudhoyono, is clearly an important step towards more effective management and conservation of the Coral Triangle area. The CTI-CFF is an inter-governmental *Agreement* among the six Coral Triangle countries that is based on a Regional Plan of Action (RPOA), which has been agreed to by all six countries, and National Plans of Action (NPOAs) that align with the RPOA, but at the same time reflecting varying national priorities. The CTI-CFF Agreement covers an area of 5.7million km^2^ that was originally biogeographically delineated by high coral diversity. Since the CTI-CFF declaration, however, it has changed also into a political agreement that covers the full exclusive economic zones of all six countries. This high-level political commitment to the CTI –CFF began when the leaders of the six countries met in Manado, Indonesia in 2009 to sign ‘The Declaration of the Regional Plan of Action’ (CTI-CFF 2009).

In its earliest stages, the CTI-CFF recognised the importance of *regional* planning in future management of the Coral Triangle area. For this to happen it was viewed as critical that government officials, managers and scientist should be able to access and visualize information and data that span national boundaries to enable national and sub-national management interventions that would have regional conservation and sustainable management impacts.

The leaders of the CT6 recognized that up-to-date spatial information is required to enable a dynamic decision making process, and to contribute to tracking progress towards RPOA and NPOA objectives. In response to these needs, the development of the CT Atlas (http://ctatlas.reefbase.org) was started in 2009 with primary funding from the US Agency for International Development through its Coral Triangle Support Partnership (CTSP). The CT Atlas is now recognized as the key tool for supporting the CTI-CFF governments and the six CTI-CFF technical working groups (TWGs). In 2012, the CT Atlas was designated by the Council of Ministers as the official database for the data storage, retrieval, and visualisation needs of the CTI-CFF.

The purpose of this paper is to describe the CT Atlas project, and the construction of the database and website, emphasising the difficulties involved and the actions so far taken to address them. We hope that the current manuscript will encourage the marine scientific community to make use of the data already collected, and actively contribute updated data, helping to ensure that the CT Atlas achieves its goal of providing a dynamic and updated decision-support tool for the CT6, development partners, research institutions, non-governmental organizations and others supporting the CTI-CFF. Ultimately this will enhance the credibility of the CT Atlas among relevant managers, scientists and policy makers in the region, leading to improved conservation and management of its natural resources.

## Methods

### Technical specifications

The CT Atlas website is hosted by ReefBase (http://www.reefbase.org) in a ‘cloud’ service provided by Amazon Elastic Compute Cloud (Amazon EC2) (http://aws.amazon.com/ec2). It uses Microsoft ASP.NET web application framework as the ‘web development tool’ with Microsoft's SQL Server as the backend database. The online interactive map section was developed using Google Map API, which supports the overlaying of various geo-referenced map images from specific sources in formats such as, Web Map Service (WMS), Keyhole Markup Language (KML) and images (PNG/GIF). Demis Web Map Server (http://www.demis.nl) delivers the map layers to the web pages. Geo-referenced ‘point’ vector data are normally served in KML format generated ‘on the fly’ from the Microsoft SQL Server; whereas the ‘polygon’ data layers are uploaded first into Demis Web Map Server and served in WMS format.

The source code was built from scratch by the team at Worldfish. We do not use a content management system like, e.g. Drupal. Some of the software used is free (e.g. Google Maps) but other parts of the system are not (e.g. MS SQL server). We are investigating whether or not to transfer CT Atlas to and Opensource system (e.g. PostgreSQL, PostGIS) but have not made the switch yet.

## Results

From the CT Atlas homepage, users can access useful links , e.g. ‘Project’, ‘Dataset’, ‘Interactive Map’, ‘MPA’ and ‘Resources’. The website content is publicly accessible, but all users must register first if they want to download GIS datasets, publications or images. Existing ReefBase (http://www.reefbase.org/) users can also use their ReefBase user account to sign in into the CT Atlas website.

### Illustrative analyses

#### Online data

A single large map of the CT Atlas region from the interactive online map section of the CT Atlas website is displayed in Figure 1. The thick red boundary is the scientific boundary of the Coral Triangle area as determined by Veron et al. [4], and is based primarily on species diversity of corals and fish. Other features which make the CT such an important marine area is the predominance of coral reefs, the variety of habitat types and their diversity, oceanography, geomorphology, bathymetry, sea level fluctuations, and river discharge [11]–[14]. The orange shapes on the map show the outlines of coral reefs in the Coral Triangle region extracted from UNEP-WCMC 2010 which is, we believe, the most up-to-date version available.

**Figure 2:**
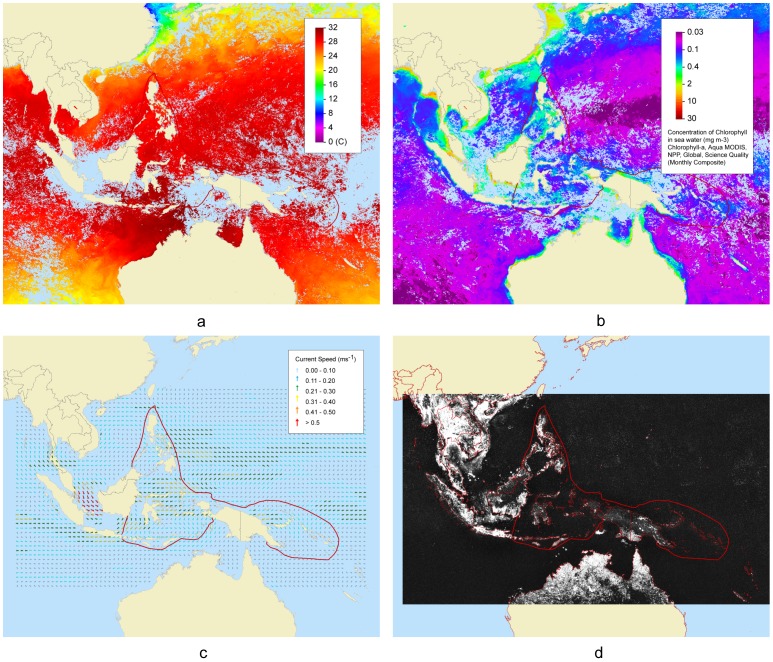
Spatial variability in selected oceanographic parameters from the Coral Triangle region. (**a**) Sea surface temperature during January 2009. The lightblue areas of sea represent locations where no data were available due to cloud coverage. (**b**) Surface chlorophyll-a concentration (mg m^−3^). The chlorophyll-a data were derived from the Moderate Resolution Imaging Spectroradiometer (MODIS; http://modis.gsfc.nasa.gov) Aqua satellite. The light blue areas of sea represent locations where no data were available due to cloud cover. (**c**) Average current speed in January 2012. Data are average (monthly, 1° grid spatial resolution) geostrophic currents derived from satellite altimetry and wind data, and were obtained from NOAA's Ocean Surface Currents Analyses, Real-Time (OSCAR; http://www.oscar.noaa.gov). (**d**) Distribution of night-lights in 2009. This map is an ‘annual composite’ which means it is an average of the highest quality night-time visible band data within each orbital swathe of the satellite. The highest quality data are free from solar and lunar illumination, cloud-cover, solar glare and auroral contamination. For these annual composites, the data are also constrained to mid-swathe where the data are less noisy and geo-referencing is more accurate. (Note: The composite product most applicable to the Coral Triangle Initiative is the normalized average lights, or “avg_lights_x_pct” product. This product is made by averaging the highest quality data, determined to be lights in the individual orbital swathes, and then normalizing this average by the percent frequency of light detection. Annual composites are generated as 30 arc-second grids and are currently available from satellites spanning the years 1992–2009).

As mentioned above, the geospatial data that we collected have been divided into categories or themes (eg. ‘Biological’ and ‘Habitats – Marine’) and sub-themes (e.g. ‘coral reefs’, ‘mangroves’, ‘seagrasses’ and ‘estuaries’) to make them easier to manage. The ‘Oceanography’ theme contains eight sub-themes, four of which (ie. ‘Sea Surface Temperature’, ‘Chlorophyll-a’ , ‘Night lights’, and ‘Current’) are highlighted here (eg. Figure 2a–d).

The only data actually collected, compiled, and updated by the team at Worldfish are the data for marine protected areas, coral bleaching, and coral disease. All the other data sets are derived, online, from other sources which are clearly credited. For the MPA data we have appointed ‘country managers’ who can log into the system directly and update the MPA data. If users note errors or problems with the data they can contact any of the Worldfish based authors directly (d.beare@cgiar.org; doug.beare@gmail.com) and any concerns will be quickly addressed.

The main downloadable product for the CT-Atlas are shapefiles which can be used in many systems, and GIS tools. The shapefiles come with metadata, in a standard format which is the Content Standard for Digital Geospatial Metadata (CSDGM), Version 2 (FGDC-STD-001-1998). This standard is, however, rather exhaustive and it was not possible to fill in every field. Spatial data quality control, validation, and metadata for the CT-Atlas is described elsewhere in detail by Cros et al. [15].

The sea surface temperature (SST) data grids in the CT Atlas come from the Pathfinder satellite (Version 5.0, http://pathfinder.nodc.noaa.gov), and are extracted directly from a web map service (http://coastwatch.pfeg.noaa.gov/erddap/wms/erdPHsstdmday/index.html). In Figure 2a, we plot SST data for January 2009 extracted from the Coral Triangle Atlas website. The map shows that SSTs range between 22°C and 32°C, and that January temperatures are highest in the eastern part of the CT region, and lowest in the north. Surface chlorophyll-*a* concentrations (mg m^−3^), which are an important indicator of aquatic primary productivity [16], are plotted for January 2013 (see Figure 2b). The graph shows comparatively low concentrations of chlorophyll-*a* in the oceanic Pacific waters east of the Philippines, and higher concentrations in all the shallower, coastal areas of the Coral Triangle region (Figure 2b): patterns one would expect due to the contrasting availability of nutrients (e.g. nitrates) between oceanic and coastal seas [17]. Ocean surface currents are displayed for January 2012 in Figure 2c. Average monthly current speed is displayed in meters per second (ms^−1^) and current direction in degrees where, for example, 90° means an eastward flowing current. The highest velocity surface current flows were observed between Sumatra and Borneo, flowing in a south-easterly direction (Figure 2c). A night light ‘annual composite’ for 2009 is plotted in Figure 2d. Night lights are potentially useful data which have, thus far, received only limited attention by the marine scientific community. On land they tend to reflect areas of dense human habitation; the brightest night lights signifying more buildings etc. In the sea, however, they summarise the distribution of boats, and ships, many engaged in commercial fishing.

### Desktop options

It is likely that the interactive online section of the website will not satisfy the users needs completely. In this instance, he or she has the option, after visualising them, to download the data, and analyse them using standard desktop GIS programs. Here we will demonstrate such an analysis by overlaying habitat types and MPA distributions obtained from the CT Atlas website.

One of the principal objectives of CT Atlas is to support the CTI-CFF Monitoring and Evaluation Technical Working Group in tracking the progress of the CTI Regional Plan of Action. This is done using ‘indicators’ designed to summarise change over time. Many of these indicators have a spatial dimension. A particularly important indicator is ‘Goal 3’ which relates to the establishment of Marine Protected Areas (MPAs), and their subsequent effective management. It thus aims to chart the gazetting of MPAs in the CT region. It is defined as: “the percentage area of total marine habitat area in the CT region that is, either in marine protected, or managed areas”.

**Figure 3:**
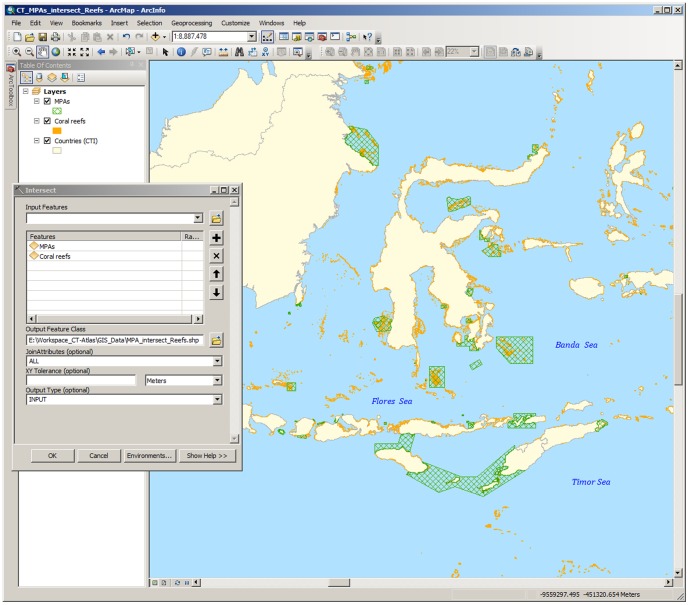
Screen shot demonstrating the overlaying of Marine Protected Area (MPA) and coral reef data from the CT-Atlas to derive the results available online in Table S1.

To demonstrate the analysis of this indicator using the CT-Atlas datasets, we downloaded the latest available for both MPAs, and the distribution of coral reefs (Figure 3). The key coral reef data layers are maintained by UNEP-WCMC (See UNEP-WCMC Ocean Data Viewer http://data.unep-wcmc.org/datasets). Hence a link from the CT-Atlas redirects users to the relevant page of UNEP-WCMC's ‘Ocean Data Viewer’, from where the data can be downloaded. For the MPA data, users must sign-in to the CT-Atlas first. Both data-sets can then be visualized and analysed simulataneously using a desktop GIS software (eg. Quantum GIS or ArcGIS).

**Figure 4:**
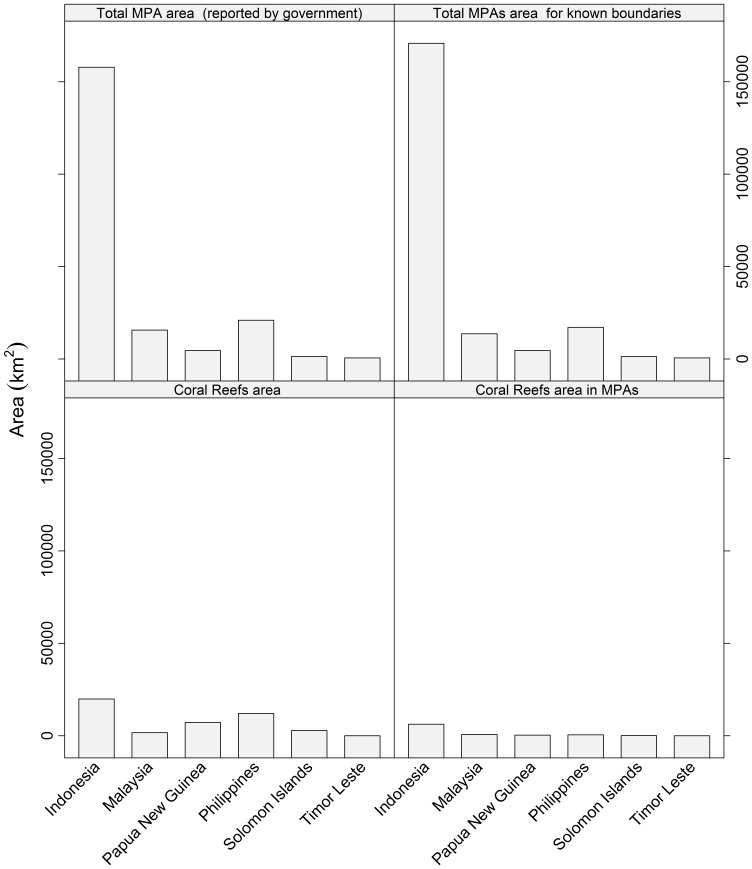
Barplot summarising: (top left) total area of Marine Protected Areas (MPAs) reported by the government; (top right) total area of MPAs with known boundaries; (bottom left) total coral reef area in each country; and (bottom right) the total area of coral reef afforded some protection by MPAs. Note data are available in the Table S1.

Most desktop GIS softwares have tools for spatial analyses, ie. ‘buffer’ zones and ‘intersections’ between different data layers can be computed. By using such tools, and simply overlaying the MPA layers on the distributions of coral reefs, it is possible to derive the total area of coral reef within the MPAs for all six CT countries (Figure 3). Figure 4 shows the results of such an analysis, see also Cros et al., 2014 [15]. The raw data are included here as supplementary material (Table S1). In terms of total area covered by MPAs, Indonesia has by far the most (*ca* 158,000 km^2^), and *ca* 31% of its coral reefs are afforded some degree of protection (Table S1).

## Discussion

The Coral Triangle Atlas (CT Atlas) is an online GIS database, providing governments, NGOs and researchers with an overview of spatial data from all six CT countries. It builds upon previous efforts to compile data at national and sub-national levels, and recognizes the pivotal role of GIS for decision-making, and effective natural resource management. Data on fisheries, biodiversity, natural resources, and socioeconomics have been collected for decades by scientists and managers working on coral reefs in general [18], [19], and on different parts of the Coral Triangle region specifically [10], [11], [20]–[23]. The CT Atlas is the first attempt, however, to collate and integrate such spatial data regionally.

In our opinion the establishment of the formal, and informal networks to enable data-sharing is the most important step in the entire process [24]. In the first stage WorldFish, together with its four partners (The Nature Conservancy, World Wildlife Fund US, Wildlife Conservation Society and IUCN), established strong ties with each other, working together to support the creation of the CT Atlas, share spatial data, and exchange methodologies, ideas and information. In the next phase of CT Atlas development, WorldFish had to establish connections with all six CT6 governments. This was done through a combination of Memoranda of Understanding (MoUs), and simpler Data Sharing Agreements, enabling the CT Atlas team to collaborate effectively with the respective agencies in all CT6 countries holding the relevant data. Without the support and help from national jurisdictions, accurate databases will never be compiled. Previously, data for the CT region were scattered, and difficult to amalgamate and examine in one place. The CT Atlas addresses this issue by compiling all the data into the same geographic formats and projections: grouping them into themes and subthemes (‘biological’, ‘geographic’ , ‘marine habitats’, ‘managed areas’, ‘oceanographic’ and ‘threats’) also makes them easier to find and manage.

The geo-spatial interactive maps are a particularly useful and important component of the website, since they showcase all the information we have collated. The data sets available are based on both vector (eg. ‘shape files’) and raster format spatial data which can be overlaid. In addition to it being possible to visualize the data layers directly with a web browser, users also have the option to search the database directly for those layers, in which they are specifically interested, download them, and use them in their preferred GIS software [25].

The development of the CT Atlas has been recognised as a focal point for the collection of geospatial data for the entire Coral Triangle region. Its vision is to improve the efficiency of management and conservation planning in the region. It will do this by giving researchers and managers access to the most complete, and most current spatial information available. It should encourage managers to share their data to complete the gaps, and reduce duplicate data collection efforts. Note: one particular data layer that could be added to the CT Atlas is the location of ongoing and completed CTI-CFF projects, and programs in the CT region, to document and track their progress, specific geographic location and scope.

The CT-Atlas is a subset of a larger global database for coral reefs known as ReefBase (http://www.reefbase.org/). The CT region has its own specific management and governance issues, and it makes sense, therefore, to package the CT data as a separate entity for managers to use. There are also specific data-sets, and layers in CT-Atlas not found on ReefBase. Scientists working on other coral reef systems around the world, e.g. the Abrolhos Bank Reef in the South Atlantic [18], [19], are free to make use of the ReefBase architecture to store and distribute their data.So, in conclusion, if the conservation management of the Coral Triangle region is actually to improve, its natural resource managers must actually *use* the data provided, and this is a significant challenge in itself. Many organisations are competing with each other to serve online data [26] and the situation is becoming ever more fragmented and chaotic. This paper, therefore, is also a plea to those collecting any data for the CT region to work with us to improve the CT Atlas, keep its databases up-to-date, and enhance its functionality.

## Supporting Information

Table S1 Summary data for coverage of legally mandated MPAs in the Coral Triangle countries (June 2013).
(DOCX)
