# Supplementary material for: The Coral Triangle Atlas: An Integrated Online Spatial Database System for Improving Coral Reef Management
Source: PLoS One. 2014 Jun 18;9(6):e96332. doi: 10.1371/journal.pone.0096332 (PMC4062393; doi:10.1371/journal.pone.0096332)
Supplement: Table S1 — Summary data for coverage of legally mandated MPAs in the Coral Triangle countries (June 2013). (DOCX) [file pone.0096332.s001.docx]

**Table S1. Summary data for coverage of legally mandated MPAs in the Coral Triangle countries (June 2013)**

|  | **INDO-NESIA** | **MALAYSIA^a^** | | | **PAPUA NEW GUINEA** | **PHILIP-PINES** | **SOLO-MON ISLAND** | **TIMORLESTE** | ***REGION*** |
| --- | --- | --- | --- | --- | --- | --- | --- | --- | --- |
| **Marine Protected Areas (MPAs)** | | | | | | | | | |
| Number of MPAs (reported by government)**^b^** | 108 | | 51 | | 59 | 1,653 | 100 | 1 | ***1,972*** |
| Total MPA area (km^2^) ( reported by government)**^b^** | 157,841 | | 15,661 | | 4,558 | 20,940 | 1,325 | 556 | ***200,881*** |
| Number MPA records in CT Atlas (point or polygon format) | 83 | | 51 | | 59 | 627 | 100 | 1 | ***920*** |
| Number of MPAs with known boundaries in CT Atlas **^c^** | 83 | | 50 | | 35 | 348 | 82 | 1 | ***599*** |
| Total MPAs area (km^2^) for known boundaries | 170,841 | | 13,653 | | 4,558 | 17,164 | 1,325 | 557 | ***208,152*** |
| **Coral Reefs ^d^** | | | | | | | | | |
| Coral Reefs area (km^2^) | 19,868 | | 1,698 | 7,256 | | 12,021 | 2,804 | 35 | ***43,682*** |
| Reefs area in MPAs (km^2^) | 6,208 | | 661 | 357 | | 471 | 113 | 10 | ***7,757*** |
| Reefs in MPAs (%) | 31.2% | | 38.9% | 4.9% | | 3.9% | 4.0% | 29.5% | ***17.8%*** |

**^a^** Malaysia MPA number and area, as well as all the calculation made include the Tun Mustapha Marine Park (10,200 km^2^) which will be fully gazetted by 2015. Pulau Yu Besar, Pulau Yu Kecil and Pulau Sipadan still have no official area reported.

**^b^** Sources for MPA data: Indonesia: Ministry of Marine Affaires and Fisheries (2013); Malaysia: Department of Marine Parks Malaysia (2009), Sabah Parks (2011), Sarawak Forestry Commission (2012); Philippines: Protected Areas and Wildlife Bureau, Laroya pers comm. (2013) thru UPMSI database; Timor Leste: Ministry of Agriculture and Fisheries (2011); no official government numbers were found for Solomon Islands and Papua New Guinea so data used is from the Coral Triangle Atlas (2013) and may slightly underestimate the number and area of MPAs in those countries.

^c^ Boundaries available through Coral Triangle Atlas (2013) as at June 2013. These boundaries were used for the rest of the calculations presented in this table.

^d^ Source of Coral Reef data UNEP-WCMC (2010).
